# Development and validation of cryopreserved or freeze-dried decellularized human dermis for transplantation

**DOI:** 10.1007/s10561-024-10131-6

**Published:** 2024-02-21

**Authors:** Giulia Montagner, Antonia Barbazza, Manvi Pant, Andrea Tancredi Lugas, Gianpaolo Serino, Cristina Bignardi, Mara Terzini, Andrea Vantini, Jacopo Stefanelli, Diletta Trojan

**Affiliations:** 1 Fondazione Banca Dei Tessuti del Veneto, Treviso, Italy; 2 University of Calgary, Calgary, Canada; 3 Politecnico Di Torino, Turin, Italy; 4 Department of Mechanical and Aerospace Engineering, Politecnico Di Torino, Turin, Italy; 5 Laboratory Service, ARPA Veneto, Verona, Italy

**Keywords:** Decellularized dermis, Allograft, Decellularization, Tissue bank, Acellular dermal matrix

## Abstract

For decades, dermal tissue grafts have been used in various regenerative, reconstructive, and augmentative procedures across the body. To eliminate antigenicity and immunogenic response while still preserving the individual components and collective structural integrity of the extracellular matrix (ECM), dermis can be decellularized. Acellular dermal matrix (ADM) products like such are produced to accurately serve diverse clinical purposes. The aim of the present study is to evaluate the efficacy of a novel decellularization protocol of the human dermis, which eliminates residual human genetic material without compromising the biomechanical integrity and collagenous content of the tissue. Moreover, a freeze-drying protocol was validated. The results showed that though our decellularization protocol, human dermis can be decellularized obtaining a biocompatible matrix. The procedure is completely realized in GMP aseptic condition, avoiding tissue terminal sterilization.

## Introduction

The human skin is a complex organ composed of the upper epidermis and lower dermis layers, which are separated by a basement membrane. The two layers differ in their vasculature, collagen content, and enzymatic potential: the superficial papillary layer is a loose connective layer that is highly vascular, while the deeper-rooted reticular layer is dense and lacks vasculature (Brown and Krishnamurthy 2022). Together the dermis layers grant support, elasticity, and tensile strength to the skin (Mason and Pham 2023).

For decades, dermal tissue grafts have been used in various regenerative (Bondioli et al. 2019), reconstructive and augmentative procedures (Ghetti et al. 2017; Bohac et al. 2018; Melandri et al. 2020) across the body. The presence of residual genetic content and cellular antigens belonging to the donor can lead to rejection, as the host recognizes these bodies as foreign, eliciting an immunogenic response carried out by host T-lymphocytes (Gilbert et al. 2009).

To combat this problem, graft tissue is decellularized with chemical reagents in order to eliminate antigenicity and immunogenic response while still preserving the individual components and collective structural integrity of the extracellular matrix (ECM). Acellular dermal matrix (ADM) products like such are produced in varying thicknesses, and derived from both xenogenic and allogenic sources to accurately serve diverse clinical purposes (Petrie et al. 2022).

A traditional and early application of ADM’s is seen in breast reconstructive and augmentative surgeries. In 2001, the potential of ADMs in breast surgery was first explored in an aesthetic capacity, during a revisional procedure (Duncan 2001). Due to their larger volume, the insertion of implants would often result in the thinning of soft tissues overlying the breast; to correct the visible rippling that occurred as a result, allograft dermis was carefully inserted (Duncan 2001). In 2005, ADMs were introduced in reconstructive surgical procedures as well (Breuing and Warren 2005); since then, it has been established that ADMs concur minimal risk, provide strength, and promote cellular repopulation, making them a great assist during breast surgery (Gravina et al. 2019).

Another frequent application of allogenic ADM’s is their assistance in wound healing. This process begins with hemostasis and inflammation of the skin, progresses through the proliferative “tissue forming” stages (epithelialization, vascularization, granulation), and terminates at maturation (Esmaeili et al. 2023).

The natural polymers that compose the ECM (collagens, elastin, proteoglycans, cell attachment factors, growth factors, laminins, polysaccharides, fibrillin, matricellular proteins and signaling molecules) make it essential for the wound healing process (Silver et al. 2017; Murphy-Ulrich and Sage 2014). Different types of collagen—which alone make up 70–80% of the ECM—are integral immunomodulators in healing, and play a supporting role in re-epithelialization and formation of granulation tissue during wound closure (Xiao et al. 2023). The ECM is often compromised in traumatic wounds such as burns, which elucidates the need for ECM rich grafts in medicine. Acellular dermal matrices (ADM’s) fill this need because they possess the healing potential of the ECM, and provide a clean and collagenous scaffold for cellular in-growth and re-vascularization (Bondioli et al. 2019).

To achieve the restorative effect without rejection, ideal ADMs should be biocompatible, mechanically resistant, non-immunogenic collagenous networks with high-suture retention (Moore and Jones 2011). To achieve this product, decellularization protocols employ physical, chemical, and enzymatic treatments (Gilbert et al. 2006).

The aim of the present study is to evaluate the efficacy of a novel decellularization protocol of the human dermis, which eliminates residual human genetic material without compromising the biomechanical integrity and collagenous content of the tissue. Moreover, a freeze-drying protocol was studied. The study was designed to fulfil the quality control requirements of the current European guide on tissue for human application (“Guide to the quality and safety of tissues and cells for human application”, EDQM, European Directorate for the Quality of Medicines and Healthcare), namely microbiological assessment, cytotoxicity tests, mechanical tests, histological evaluation, DNA quantification and residual water of freeze-dried dermis.

## Methods

### Tissue procurement

Human dermis was procured from cadaver donor following Italian directives and with the proper informed consent. Three batches of dermis, obtained from three different donors and unsuitable for transplantation but morphologically unaltered were utilized for the protocol set up and validation. The retrievals were performed using a dermatome (Aesculap Inc, USA), within 24 h of cardiac arrest or 12 h if the cadavers were not refrigerated during the first six hours after death. Dermis patches were retrieved from the back. After retrieval, the tissues were transferred in BASE medium (Alchimia srl, Italy) containing gentamicin 200 µg/ml (Fisiopharma, Italy), vancomycin 100 µg/ml (Pharmatex, Italy) and meropenem 200 µg/ml (Fresenius Kabi AG, Germany), a solution validated for tissues decontamination (Serafini et al. 2016, Paolin et al. 2017, Montagner et al. 2018). Tissues were subsequently transported to the tissue establishment at + 4 °C.

### Tissue process before decellularization

After of the first decontamination, dermis was processed in grade A cabinet flow hood in grade B laboratory. Tissues were decontaminated for the second time in BASE medium (Alchimia srl, Italy) and gentamicin 200 µg/ml (Fisiopharma, Italy), vancomycin 100 µg/ml (Pharmatex, Italy) and meropenem 200 µg/ml (Fresenius Kabi AG, Germany)for minimum 48 h. At the end of the second decontamination, dermis patches were packaged in double sterile ethylene vinyl acetate (EVA) bags (Agricons Ricerche, Italy) and stored at -80 °C until the beginning of decellularization. The temporary storage at −80 °C lasted an average of ten days.

### Thawing before decellularization

Before decellularization, dermis patches were thawed immersing their containers in warm water (37 °C). Tissues were then washed in sterile saline solution.

### Decellularization

Dermis patches decellularization were carried out in three consecutive days. The protocol is patent pending and is based on a hypertonic solution and two reagents, benzonase (Sigma-Aldrich, USA) and sodium cholate (Sigma-Aldrich, USA). At the end of the decellularization, dermis patches were transferred in BASE medium (Alchimia srl, Italy) containing gentamicin 200 µg/ml (Fisiopharma, Italy), vancomycin 100 µg/ml (Pharmatex, Italy) and meropenem 200 µg/ml (Fresenius Kabi AG, Germany) overnight. The following day, tissues were cryopreserved or freeze-dried.

### Microbiological analysis

Several microbiological tests were established throughout the dermis process in order to verify the compliance with the acceptance criteria and regulation. Tissue samples were tested for bioburden before processing and at the end of decellularization and decontamination, immediately before final packaging. Bioburden test was carried out by a microbiology, following their own validated procedure and material. Liquid samples of the solutions that were in contact with the tissueduring processing were inoculated and incubated in BD BACTEC culture vials, in accordance with the manufacturer’s instructions (BD, Becton, Dickinson and Company, USA). If the samples tested positive, the microorganisms were isolated and identified using standard procedures. Finally, environmental microbiological monitoring was conducted at each step of the process.

### Cryopreservation

Before cryopreservation, each dermis patch was transferred in ethylene vinyl acetate (EVA) bags (Agricons Ricerche, Italy) and immersed in a solution of BASE medium (Alchimia, Italy), dimethyl sulfoxide (WAK-Chemie Medical GmbH, Germany) and human albumin (Kedrion S.p.A., Italy). Cryopreservation was achieved using a programmable cryogenic freezer (Planer KryoSave Integra 750–30, Planer Limited, UK), which triggered a controlled cooling rate. Dermis patches were then stored in vapor-phase liquid nitrogen. Cryopreserved samples were analysed for the content of collagen and mechanical tests.

### Freeze-drying

Before freeze-drying, each dermis patch was transferred on a tray and inserted in a Tyvek/OPA bag (Encaplast, Italy) that was subsequently sealed. Freeze-drying runs were performed at a Scientific Products (USA) freeze-dryer. The cycle lasted about 24 h and was made up of 17 steps. The temperature range during freeze-drying was between -30 °C and 65 °C and the vacuum was set between 50 and 150 mTorr.

### Histological analysis and fluorescence staining of nuclei

Samples of dermis (native and decellularized) were embedded in Optimal cutting temperature compound (OCT compound, Kaltek, Italy) using PrestoCHILL (Milestone Medical, Italy). Tissue blocks were then sliced into 6 µm sections, which were fixed in 95% ethanol, rehydrated with 70% ethanol and stained with haematoxylin and eosin (Merck, Germany). For nuclei fluorescence staining, tissue sections were fixed in ethanol 95% and stained with Hoechst 33,342 (Thermo Fisher Scientific, USA). Pictures were taken using the light and fluorescent Leica DMi8 microscope equipped with camera (Leica Microsystems, Germany).

### DNA residual analysis

After freeze-drying, 10 mg of each sample was weighed for DNA extraction, which was achieved using QIAamp DNA Mini Kit (Qiagen, Germany), following manufacturer’s instructions. DNA concentrations were measured with Qubit 4 fluorometer (Thermo Fisher Scientific, USA), using the Qubit™ 1X dsDNA High Sensitivity (Thermo Fisher Scientific, USA). Moreover, the presence of DNA fragments was observed by gel electrophoresis, using the GeneRuler Express DNA Ladder (Thermo Fisher Scientific, USA) as a marker.

### Collagen quantitative analysis

After thawing, each sample was weighed and digested with 0.1 mg/ml pepsin (Sigma-Aldrich, USA) in 0.5 M acetic acid (Merck, Germany). Collagen quantification was performed by Sircol S1000 assay (Biocolor, UK) following manufacturer’s instruction. Quantification was performed using the Byonoy absorbance microplate reader (Byonoy GmbH, Germany). The detection range was 0–150 µg/ml.

### In vitro* citotoxicity tests*

*Test by *direct* contact*

A standard contact cytotoxicity test (ISO 10993–5:2009 Biological evaluation of medical devices—Part 5: Tests for in vitro cytotoxicity) was conducted against human fibroblast cells (BJ CRL-2522, ATCC, USA). Briefly, cells were seeded at 25,000 cell/cm^2^ in Minimum Essential Medium Eagle (MEM, Thermo Fisher Scientific, USA), 10% (v/v) Fetal Bovine Serum (FBS, Thermo Fisher Scientific, USA), 1% (v/v) Penicillin/Streptomycin (Thermo Fisher Scientific, USA) and incubated at 37 °C in an atmosphere of 5% CO_2_. Cell growth in absence (negative control) or presence of decellularized dermis was visualized with Giemsa stain and observed using Leica DMi8 microscope equipped with camera (Leica Microsystems, Germany). Cyanoacrylate glue was used as positive control. Reactivity was evaluated according to ISO 10993–5:2009.

*Test on extracts*

Cells were seeded at 15,000 cells/cm^2^ in MEM (Thermo Fisher Scientific, USA), 10% (v/v) FBS, (Thermo Fisher Scientific, USA), 1% (v/v) Pen/Strep (Thermo Fisher Scientific, USA) and incubated at 37 °C in an atmosphere of 5% CO_2_ until the cells reached 50% confluency. Culture media was preconditioned with 0,1 g/ml and 0,2 g/ml of decellularized dermis. Cells were then treated with preconditioned media for 48–72 h. Cells viability was evaluated with MTT test (3-(4,5-dimethylthiazol-2-yl)-2,5-diphenyltetrazoliumbromid, Thermo Fisher scientific, USA) using the Byonoy absorbance microplate reader (Byonoy GmbH, Germany). According to ISO 10993–5:2009, reduction of cell viability by more than 30% was considered a cytotoxic effect.

### Mechanical tests

Uniaxial tensile tests were performed to investigate the mechanical properties of the dermis. Three decellularized and three non-decellularized patches (cryopreserved), obtained from three different donors, were tested. Prior mechanical testing, each patch was immersed in water at 37 °C including packaging until the conservation medium was completely melted. Then, 16 rectangular specimens (length 40 mm, width 5 mm) were cut from each patch using a custom cutting tool. A total of 96 specimens (48 decellularized, 48 non-decellularized) were obtained. The thickness was measured with a micrometre in three points of the central region of each specimen, and the average of the three measurements was taken as the specimen thickness.

**Fig. 1:**
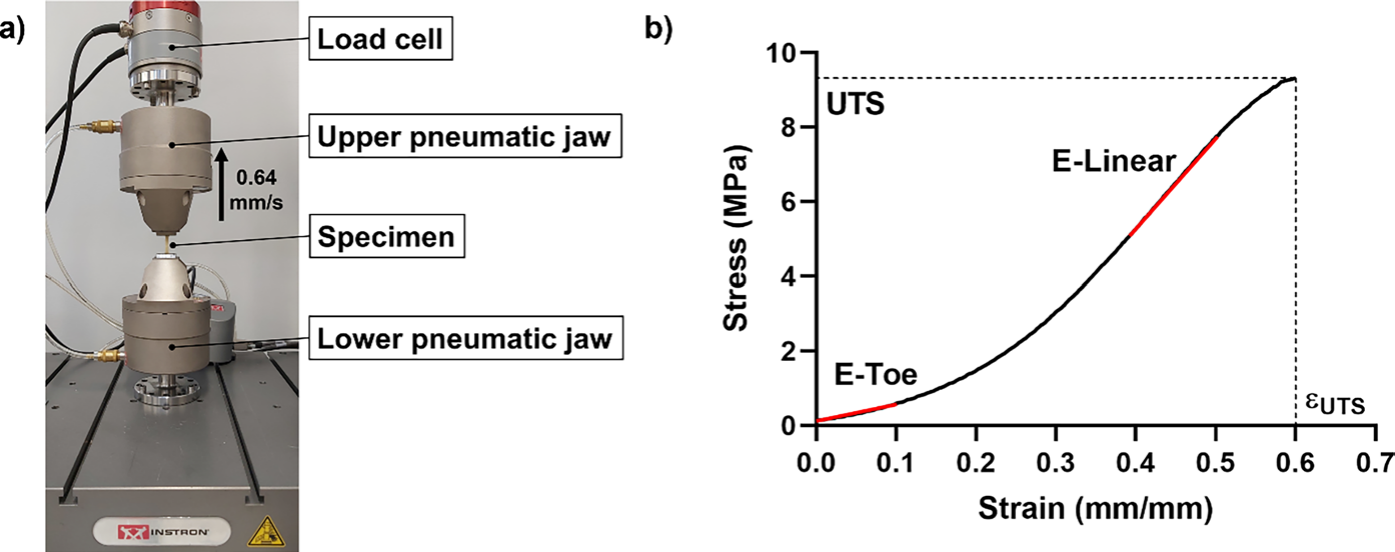
Uniaxial tensile tests setup (**a**). Mechanical parameters extracted from a typical stress–strain curve (**b**)

A universal testing machine (Instron E3000, USA) was used for the uniaxial tensile tests. A length of 10 mm at each end of the specimen was gripped in a pneumatic jaw as depicted in Fig. 1a, thus obtaining a 20 mm gauge length. The specimens were loaded at a constant rate of 0.64 mm/s—i.e., 3.2% of the gauge length per second (Terzini et al. 2016)—until failure, while measuring the load applied on the specimens with a 3 kN load cell.

Figure 1b shows an explanatory stress–strain curve as obtained for each tested sample. The stress and the strain were computed as described in Eq. 1 and Eq. 2.

1 $$\sigma =\frac{F}{t\times w}$$

2 $$\varepsilon = \frac{l-{l}_{0}}{{l}_{0}}$$

With the following terms meanings:

- $$\sigma$$ = stress (MPa)
- $$F$$ = measured force (N)
- $t$ = specimen thickness (mm)
- $w$ = specimen width (mm)
- $\varepsilon$ = strain (mm/mm)
- $l$ = specimen instantaneous gauge length (mm)
- ${l}_{0}$ = specimen initial gauge length (mm)

From the stress–strain curves, which characterize the mechanical behaviour of the tested samples, four parameters were evaluated: the ultimate tensile strength (UTS, i.e., the stress at failure), the elongation at break (εUTS, i.e., the strain at failure), the toe elastic modulus (E-Toe, i.e., the slope of the stress–strain curve between 0 and 10% strain), and the linear elastic modulus (E-Linear, i.e., the slope of the stress–strain curve in its linear region).

### Residual moisture analysis

The analysis was performed in three batches of decellularized freeze-dried dermis. A sample of dermis was weighted before and after drying it in an oven at 103 ± 2 °C overnight. The residual moisture content was obtained from the ratio between the weight lost after drying and the initial weight.

### Statistical analysis

In DNA and collagen quantification the t single sample test was used and *p* values less than 0.05 (*p* < 0.05) were considered statistically significant.

Two-sample t-test were used to compare mechanical parameters of decellularized vs native dermis, and *p* values less than 0.05 (*p* < 0.05) were considered statistically significant.

## Results

### Microbiological analysis

All the microbiological tests were compliant with the acceptance criteria. Dermis patches were effectively decontaminated, even without terminal sterilization. All the environmental controls were negative, demonstrating that the entire process is achievable in a Good Manufacturing Practice (GMP) compliant facility and as a tissue bank practice.

### Histological evaluation

**Fig. 2:**
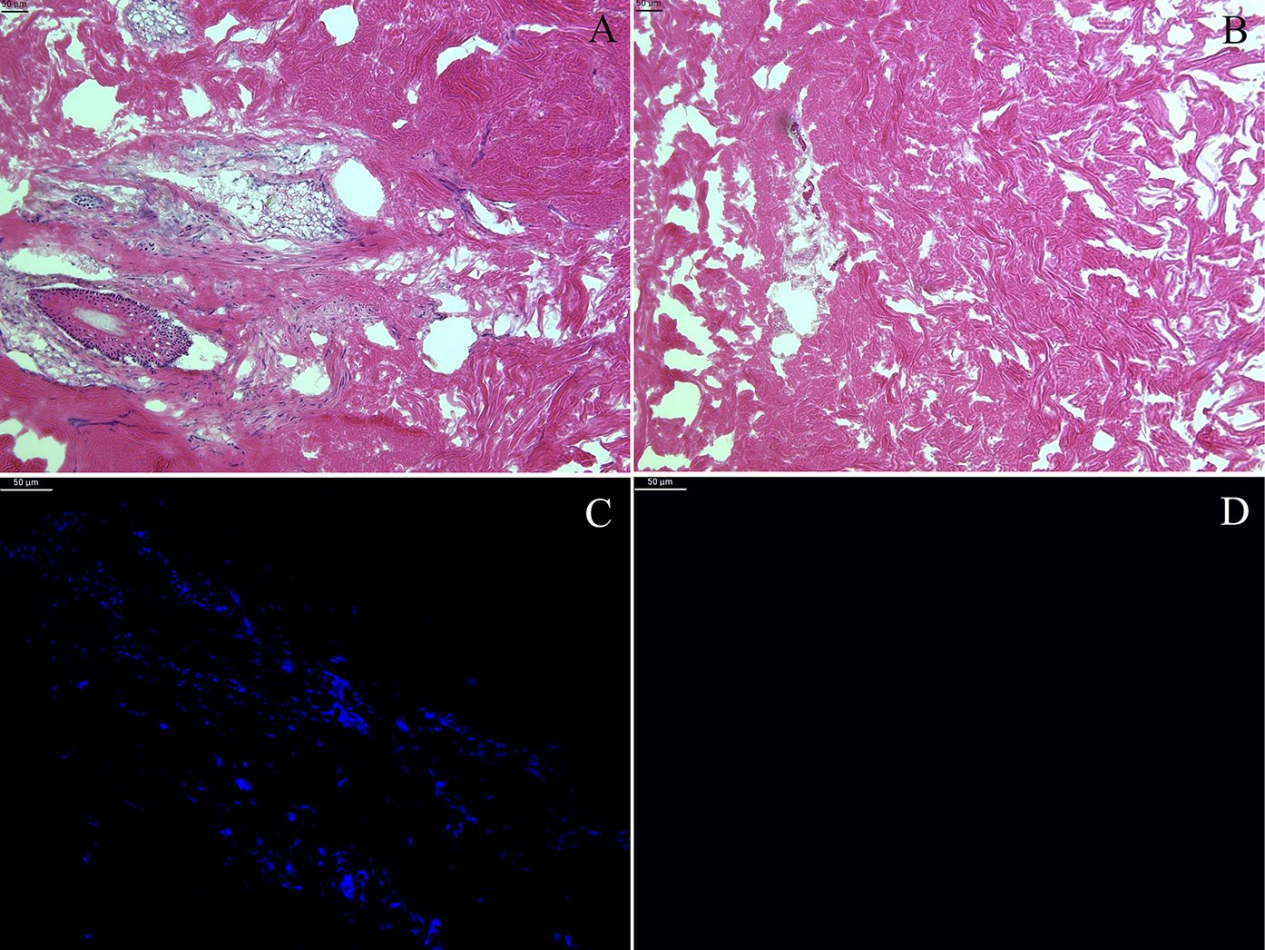
Haematoxylin and eosin staining of representative histological sections of non-decellularized dermis (**A**) and decellularized dermis (**B**), 10 × magnification. Absence of nuclei is demonstrated in decellularized samples. Extracellular matrix is similar between non-decellularized dermis (**A**) and decellularized dermis (**B**). Nuclear fluorescence staining with Hoechst in representative samples of non-decellularized dermis (**C**) and decellularized dermis (**D**). Nuclei are absent in decellularized sample. 20 × magnification

Haematoxylin and eosin staining of dermis demonstrated the maintenance of the extracellular matrix in decellularized samples compare to non-decellularized ones (Fig. 2). Nuclei were absent in decellularized sample, while they were identifiable in native samples, as demonstrated both by haematoxylin and eosin staining and Hoechst staining (Fig. 2).

### DNA residual analysis

**Fig. 3:**
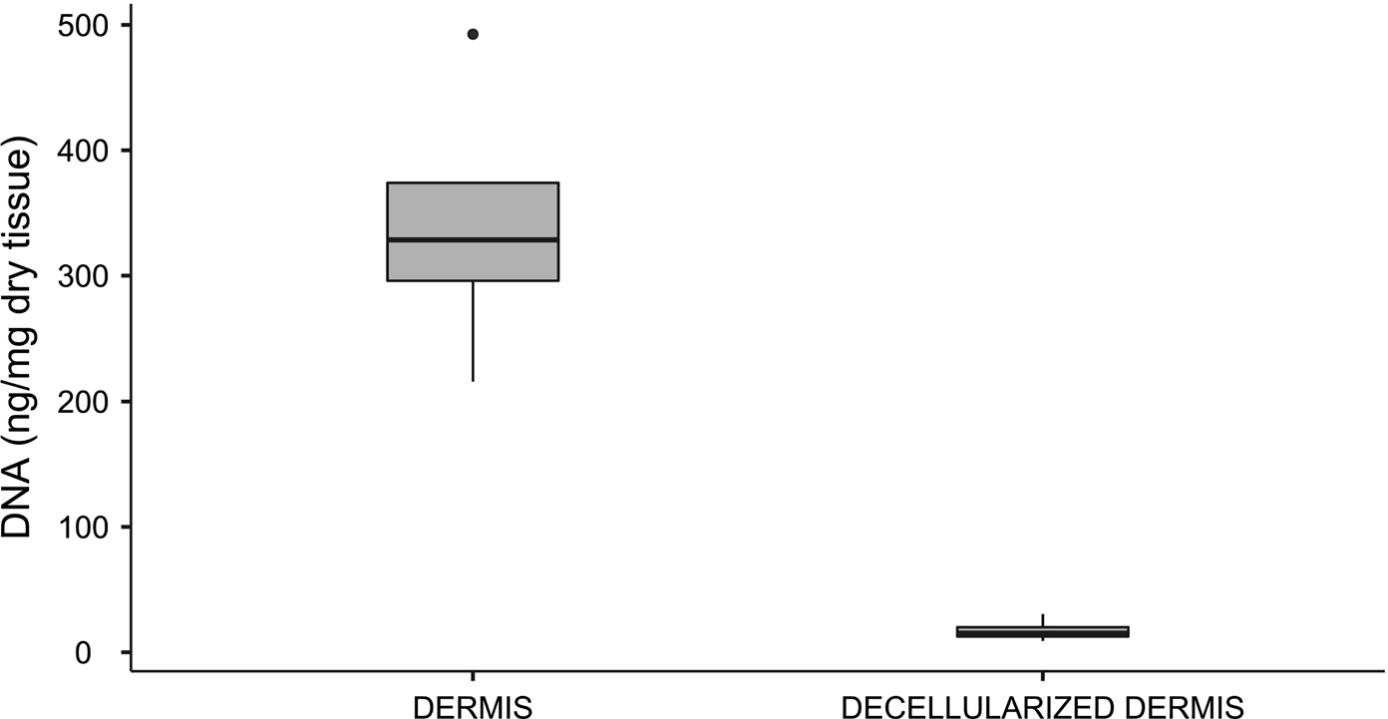
DNA content in four batches of dermis (native and decellularized) obtained from four different donors. The residual DNA in decellularized dermis was reduced to less than 10% with respect to the DNA quantity of native dermis; the reduction depicted is statistically significant (*p* < 0.05)

**Fig. 4:**
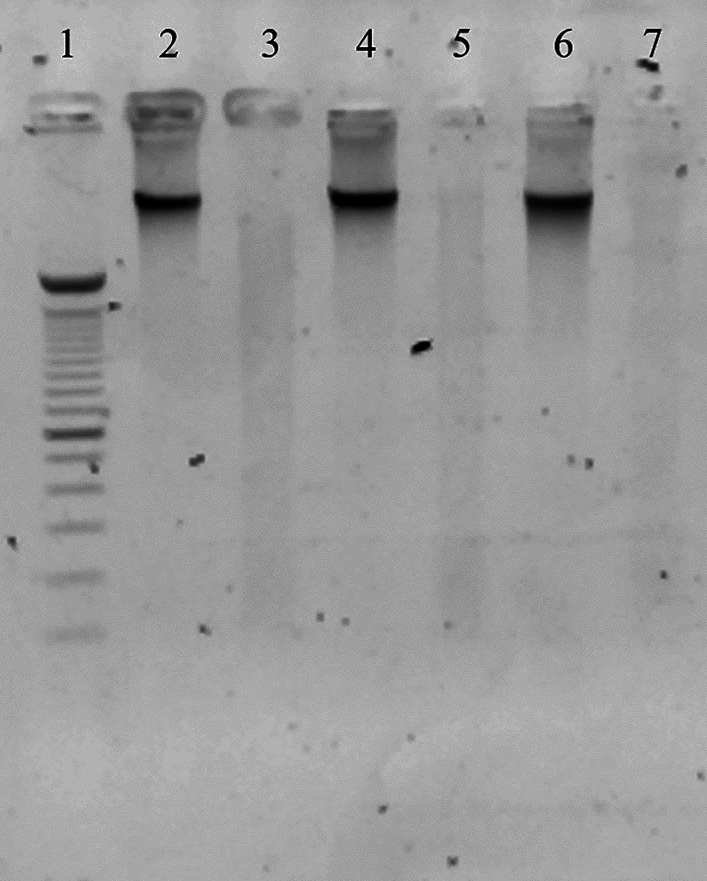
Electrophoresis analysis of nuclei acids. The image demonstrates the presence of DNA in non-decellularized samples (2, 4, 6) and the absence of nucleic acids fragments in decellularized samples (3, 5, 7). Samples were compared to a marker (1)

The quantification of residual DNA detected a reduction of 90% of DNA in decellularized samples, that contained less than 50 ng of DNA/mg of dry tissue as required for an efficient decellularization (Crapo et al. 2011). Figure 3 reports the DNA quantity of decellularized and non-decellularized samples of four batches of dermis. Moreover, electrophoresis analysis demonstrated the absence of nucleic acids fragments in decellularized samples (Fig. 4).

### Collagen quantitative analysis

The quantification of collagen demonstrated that the decellularization did not alter the extracellular matrix; in the three different batches of dermis analysed (three different donor), no statistical reduction of collagen content was observed in decellularized samples compared to non-decellularized samples of the same batch (Table 1).

**Table 1:** Collagen content obtained from the analysis of three batches of dermis, native and decellularized. No reduction of the extracellular matrix component was revealed

	Collagen content	
	Non-decellularized samples (µg/mg)	Decellularized samples (µg/mg)
Batch 1	1.56 ± 0,0205	3.32 ± 0,1062
Batch 2	2.37 ± 0,0849	3.87 ± 1,4173
Batch 3	1.72 ± 0,0187	2.41 ± 0,1334

### In vitro* cytotoxicity tests*

**Fig. 5:**
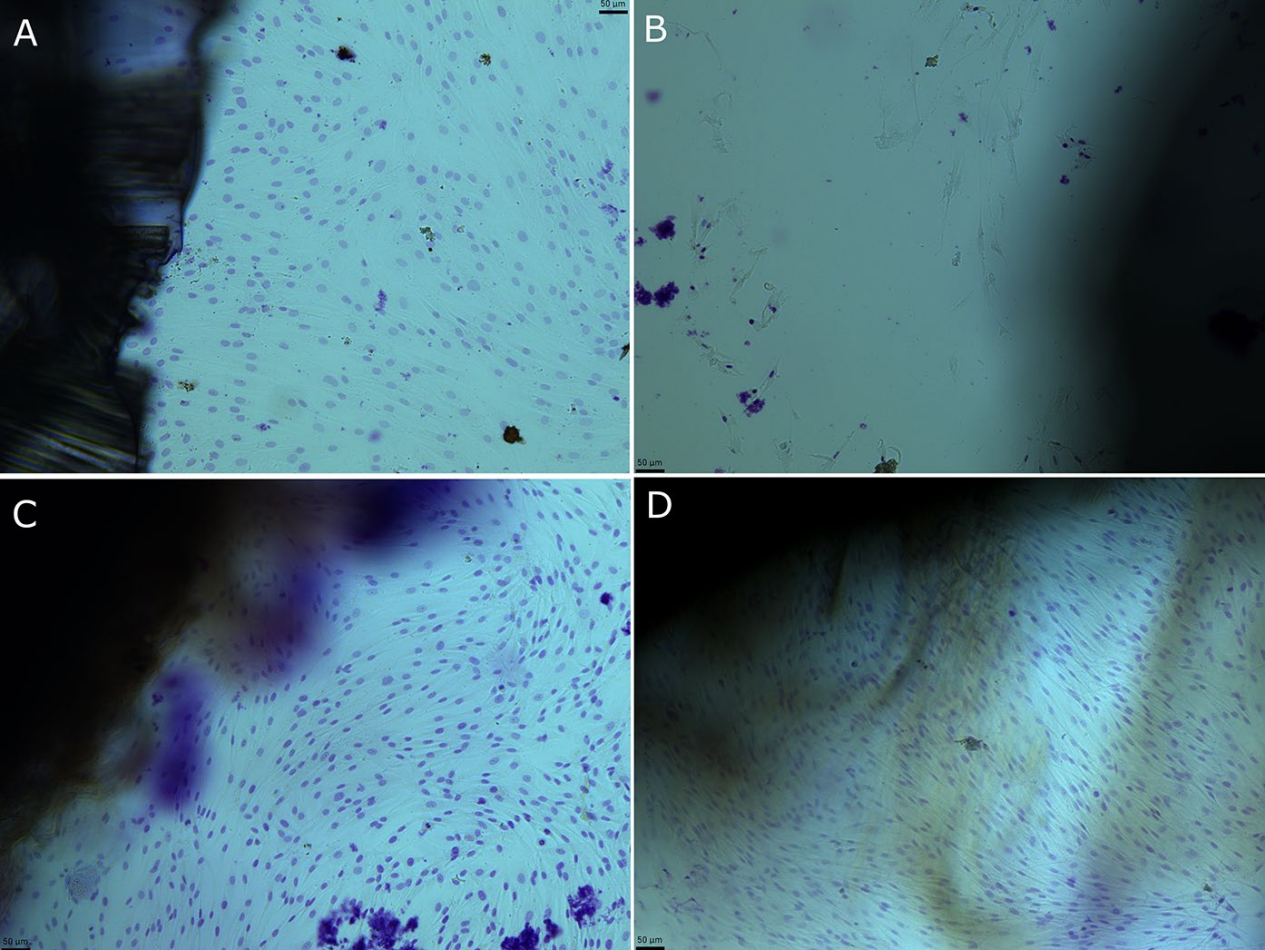
Representative figures of contact cytotoxicity test. Fibroblasts BJ CRL-2522 were grown in absence of cytotoxic agent (**a**), in presence of a cytotoxic agent (**b**, cyanoacrylate glue), in presence of non-decellularized dermis (**c**) or decellularized dermis (**d**). Cells were visualized with Giemsa stain at light microscopy and 10 × magnification

**Fig. 6:**
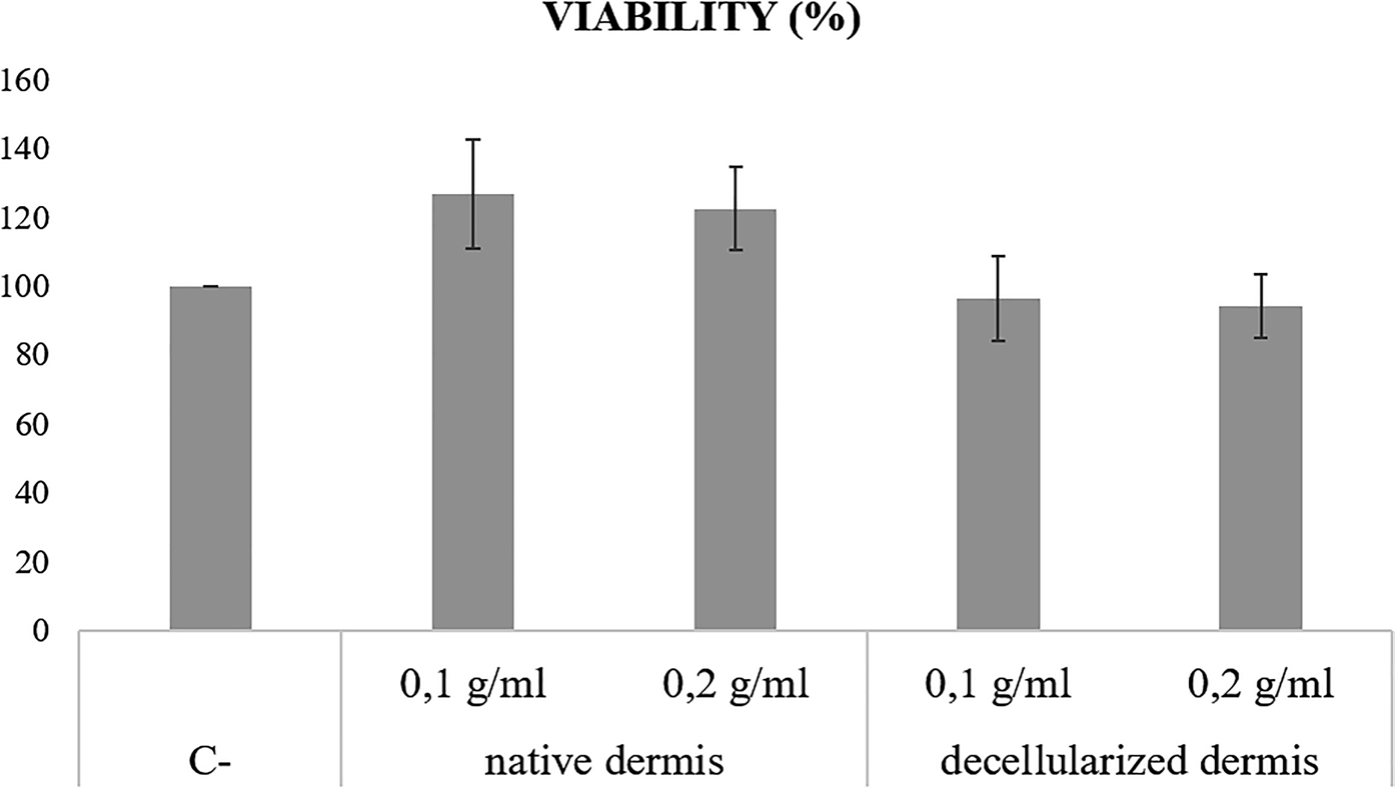
Viability of fibroblasts BJ CRL-2522 cultured in preconditioned medium with 0,1 g/ml and 0,2 g/ml of native dermis or decellularized dermis. Three batches of both native and decellularized samples obtained from three different donors were tested. No cytotoxicity occured, since any reduction of cell viability by more of 30% was observed

No reactivity was observed in cells cultures added with decellularized dermis, while inhibition occurred in the positive controls (Fig. 5). Moreover, the test on extract revealed the maintenance of cells viability as depicted in Fig. 6. According to ISO 10993-5:2009, no cytotoxic activity was observed since any reduction by more than 30% occurred.

### Mechanical tests

The mean thickness of the specimens for each dermis patch is shown in Table 2.

**Table 2:** Thickness of the specimens (mean ± SD)

	Non-decellularized samples	Decellularized samples
Batch 1 (mm)	3.47 ± 0.28	2.91 ± 0.14
Batch 2 (mm)	2.83 ± 0.46	3.18 ± 0.10
Batch 3 (mm)	3.25 ± 0.12	3.02 ± 0.19

**Fig. 7:**
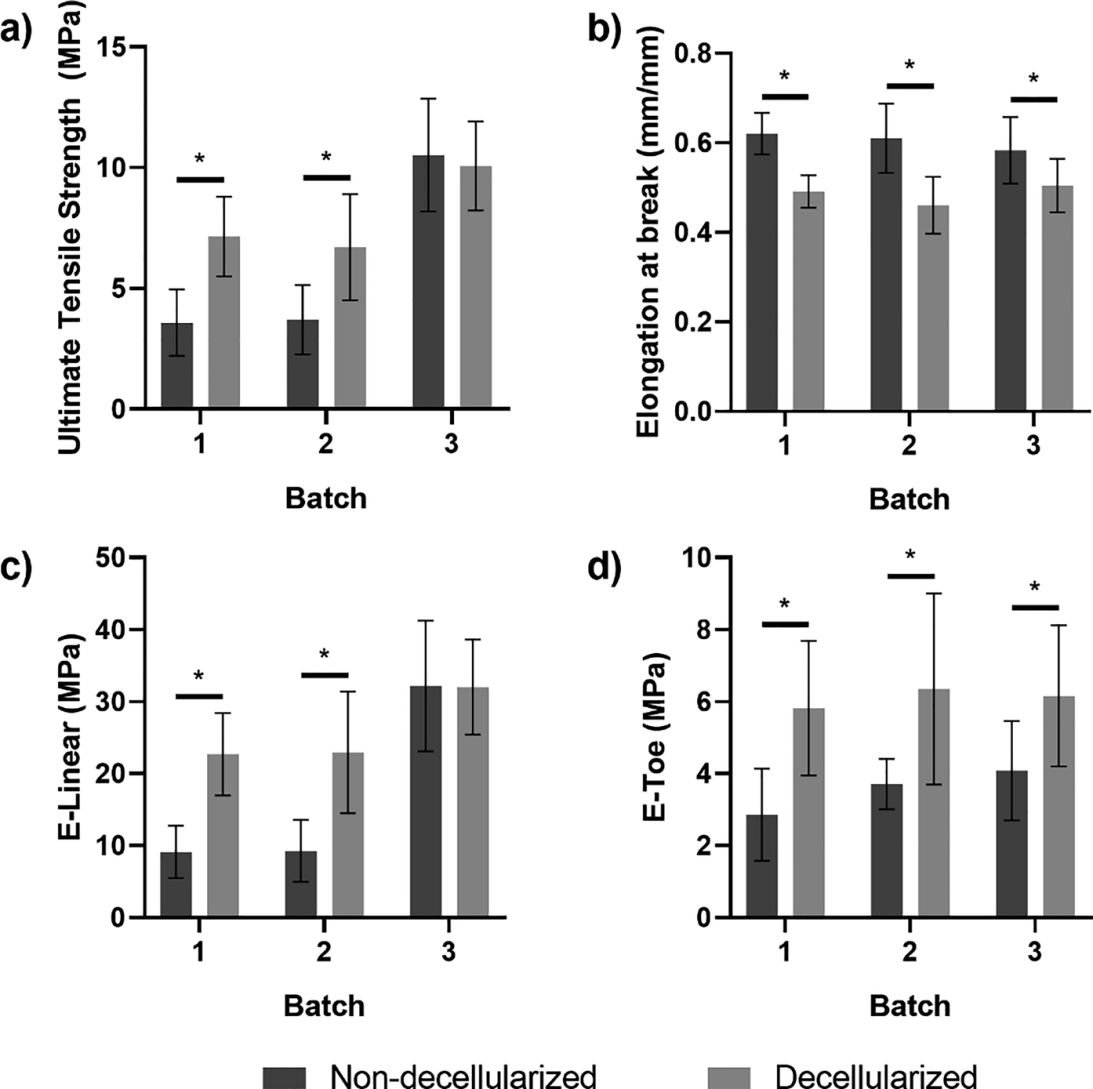
Mechanical tests results: ultimate tensile strength (**a**), elongation at break (**b**), linear elastic modulus (**c**), and toe elastic modulus (**d**). Asterisks above bars indicate statistically significant differences (*p* < 0.5)

The ultimate tensile strength obtained for decellularized patches resulted significantly higher than the UTS of the non-decellularized ones in batches 1 and 2 (*p* < 0.05). Instead, no significant difference was highlighted in batch 3. All batches of non-decellularized dermis showed a significantly higher elongation at break compared to the decellularized ones (*p* < 0.05) while the toe elastic modulus of the treated (decellularized) dermis resulted significantly higher than the control (non-decellularized) patches (*p* < 0.05) in all batches. The linear elastic modulus was significantly higher in treated patches compared to control ones in batch 1 and batch 2 (*p* < 0.05). Instead, no significant differences were found for batch 3. All the results are summarized in Fig. 7 and Table 3.

**Table 3:** Mechanical tests results summary (mean ± SD)

		Non-decellularized	Decellularized	Difference
Batch 1	UTS (MPa)	3.58 ± 1.38	7.15 ± 1.38	99.72%
	E-Toe (MPa)	2.86 ± 1.28	5.28 ± 1.87	84.62%
	E-Linear (MPa)	9.12 ± 3.64	22.72 ± 5.74	149.12%
	ε_UTS_ (mm/mm)	0.62 ± 0.05	0.49 ± 0.04	-20.97%
Batch 2	UTS (MPa)	3.71 ± 1.44	6.71 ± 2.20	80.86%
	E-Toe (MPa)	3.71 ± 0.70	6.35 ± 2.66	71.16%
	E-Linear (MPa)	9.28 ± 4.28	22.96 ± 8.44	147.41%
	ε_UTS_ (mm/mm)	0.61 ± 0.08	0.46 ± 0.06	-24.59%
Batch 3	UTS (MPa)	10.52 ± 2.35	10.07 ± 1.84	-4.28%
	E-Toe (MPa)	4.08 ± 1.38	6.17 ± 1.96	51.23%
	E-Linear (MPa)	32.20 ± 9.08	32.04 ± 6.59	-0.50%
	ε_UTS_ (mm/mm)	0.58 ± 0.07	0.50 ± 0.06	-13.79%

### Residual moisture

This analysis revealed that the freeze-drying method was efficient since the residual moisture of the three batches of freeze-dried decellularized dermis was below the limit of 5% recommended by the European guide (“Guide to the quality and safety of tissues and cells for human application”, European Directorate for the Quality of Medicines and Healthcare).

## Discussion

In recent years, acellular dermal matrices have become popular for many clinical applications, mainly for wound healing, breast reconstruction, rotator cuff repair. This study reports the analysis performed on a human dermis decellularized with a new protocol. Histology and DNA residual analysis demonstrated the removal of the genetic material from the tissue after decellularization. Perez et al., who recently published a fast decellularization protocol of human dermis, reported a similar reduction of DNA (Perez et al. 2021), but the post-decellularization DNA content of commercially available ADMs is noticeably higher (Moore et al. 2015). While other authors investigated the alteration of collagen IV after dermis decellularization, we demonstrated the maintenance of the total collagen content after decellularization, similarly to what has been reported by Perez et al. (Perez et al. 2021). Cytotoxicity tests revealed no toxic activity on cultured fibroblasts. Regarding the mechanical properties of the decellularized tissue, the average values summarized in Table 3 highlighted different properties between treated and control patches in batches 1 and 2. In particular, the treated tissue resulted stiffer since the E-Toe and the E-Linear were found to be significantly higher than the control tissue. Moreover, after the treatment, the examined tissue was able to bear higher values of stresses (higher values of UTS) before failing, but the elongation at break decreased, indicating a reduced capability of the tissue to bear deformations. Specimens derived from batch 3 showed a higher linear elastic modulus and UTS compared to the other batches, although the differences between control and treatment were less evident.

Although previous works highlighted a decrease of UTS and elastic modulus after human dermis decellularization based on incubation in 0.06 N NaOH or DMEM for 1–7 weeks (Terzini et al. 2016), other findings confirmed the possibility of an increase of the same properties in treated tissue compared to native one. Bondioli et al. (Bondioli et al. 2014) obtained a UTS increase of 88.89% and an elastic modulus increase of 86.36% after decellularization (overnight incubation in 2.5% trypsin 10 × , diluted tenfold), which is comparable with the results here obtained. Although no comparisons with native tissue were found in literature, the findings presented in this study are in agreement with those found in the analysis carried out by Bottino et al. which performed uniaxial tensile tests on AlloDerm®, a widely accepted acellular dermal matrix (ADM) for soft tissue applications. Taking into account the inherent variability of biological tissues intra/inter-subject and the lacking information about the harvesting orientation, as well as the relative spatial arrangement of the patches in the donor back, the results shown that the treatment improved the capability of the analysed tissues to bear higher level of stresses with respect the control tissues but, as side effect, partially reduced their elongation properties.

Our study showed that though our decellularization protocol, human dermis can be decellularized in aseptic condition, avoiding tissue terminal sterilizationand through a feasible procedure for a tissue bank. The results obtained demonstrate the compliance with the quality control requirements of the current European guideline. Further studies would be necessary to assess in vivo immunogenicity.
